# Exploring Strategies to Prevent and Treat Ovarian Cancer in Terms of Oxidative Stress and Antioxidants

**DOI:** 10.3390/antiox14010114

**Published:** 2025-01-20

**Authors:** Yanru Long, Houhui Shi, Jiatian Ye, Xiaorong Qi

**Affiliations:** Key Laboratory of Birth, Defects and Related Diseases of Women and Children, Department of Gynecology and Obstetrics, Ministry of Education, West China Second Hospital, Sichuan University, Chengdu 610041, China; long_yr@163.com (Y.L.); shihouhui@foxmail.com (H.S.); yejiatian@stu.scu.edu.cn (J.Y.)

**Keywords:** ovarian cancer, oxidative stress, ROS, RNS, antioxidants, treatment, prevention

## Abstract

Oxidative stress is a state of imbalance between the production of reactive oxygen species (ROS) and reactive nitrogen species (RNS) and the antioxidant defence system in the body. Oxidative stress may be associated with a variety of diseases, such as ovarian cancer, diabetes mellitus, and neurodegeneration. The generation of oxidative stress in ovarian cancer, one of the common and refractory malignancies among gynaecological tumours, may be associated with several factors. On the one hand, the increased metabolism of ovarian cancer cells can lead to the increased production of ROS, and on the other hand, the impaired antioxidant defence system of ovarian cancer cells is not able to effectively scavenge the excessive ROS. In addition, chemotherapy and radiotherapy may elevate the oxidative stress in ovarian cancer cells. Oxidative stress can cause oxidative damage, promote the development of ovarian cancer, and even result in drug resistance. Therefore, studying oxidative stress in ovarian cancer is important for the prevention and treatment of ovarian cancer. Antioxidants, important markers of oxidative stress, might serve as one of the strategies for preventing and treating ovarian cancer. In this review, we will discuss the complex relationship between oxidative stress and ovarian cancer, as well as the role and therapeutic potential of antioxidants in ovarian cancer, thus guiding future research and clinical interventions.

## 1. Introduction

Ovarian cancer is a common and non-negligible type of gynaecological malignancy, which seriously affects women’s health [1]. According to statistics, there are about 313,959 new cases of ovarian cancer and 207,252 deaths worldwide in 2020 [2]. Thus, ovarian cancer affects women’s health and lives worldwide and is a category of disease that needs to be explored and researched in depth. Ovarian cancer can be seen in women of any age and includes multiple types, such as epithelial carcinoma, malignant germ cell tumour, and malignant gonadal mesenchymal tumour, and epithelial carcinoma is the most common [3]. Ovarian epithelial carcinoma is further classified into plasma carcinoma, endometrioid carcinoma, clear cell carcinoma, and mucinous carcinoma [4]. Most ovarian cancers are difficult to detect because they are asymptomatic in the early stages, making it challenging to achieve an early diagnosis [5]. Clinically, it has been found that most of the patients are in advanced stages when they are diagnosed, and it is obvious that treatment is ineffective for these patients [6]. In clinical practice, the treatment of ovarian cancer is mainly based on surgery, combined with chemotherapy, targeted therapy, and immunotherapy [7]. However, it has been found that the recurrence rate and mortality rate of ovarian cancer are still high after treatment. Obviously, the diagnosis and treatment of ovarian cancer are still problems difficult to overcome. Therefore, more effort should be devoted to the development of new strategies for the treatment of ovarian cancer.

Oxidative stress arises when there is an imbalance between oxidative and antioxidant effects [8]. With the in-depth exploration of oxidative stress, a number of studies have found that oxidative stress may cause damage to cells and tissues, which affects the whole organism and leads to the occurrence of various diseases, such as diabetes, malignant tumours, Alzheimer’s disease, and Parkinson’s disease [9,10]. In addition, oxidative stress is not only related to the development of cancer but also closely connected with the invasion and metastasis of cancer [11]. In recent years, some studies have found that oxidative stress also plays an important role in ovarian cancer. On the one hand, a certain range of ROS promotes the proliferation and metastasis of ovarian cancer, while on the other hand, a high level of ROS can induce apoptosis in ovarian cancer cells through its cytotoxic effects [12]. Hence, oxidative stress in ovarian cancer may be considered as a direction to explore therapeutic modalities. For example, in some cases, inhibiting oxidative stress might hinder the invasion and metastasis of ovarian cancer [13]. Importantly, antioxidants, an indispensable part of the oxidative stress process, may influence the prevention and treatment of ovarian cancer [14]. Therefore, exploring oxidative stress and antioxidants may be a worthwhile new direction in the treatment of ovarian cancer.

This review will analyse the relationship between oxidative stress and the development of ovarian cancer and try to explore new clinical approaches to the prevention and treatment of ovarian cancer in terms of oxidative stress and antioxidants.

## 2. Relationship Between Oxidative Stress and Ovarian Cancer

There is a complex relationship between oxidative stress and ovarian cancer, and the two interact to influence the development and progression of ovarian cancer.

### 2.1. Oxidative Stress

Oxidative stress occurs when the body is unable to maintain the normal balance between oxidation and antioxidant effects [15]. In this case, reactive oxygen species (ROS) and reactive nitrogen species (RNS) accumulate excessively in the cell or organism, and a large number of oxidative intermediates are produced, such as hydrogen peroxide, malondialdehyde, etc. Oxidative stress can be affected not only by metabolic disorders and inflammatory responses in vivo but also by exogenous factors such as radiation and drugs [16]. Oxidative stress will be described below in terms of ROS and RNS.

#### 2.1.1. Definition and Sources of ROS

**Figure 1:**
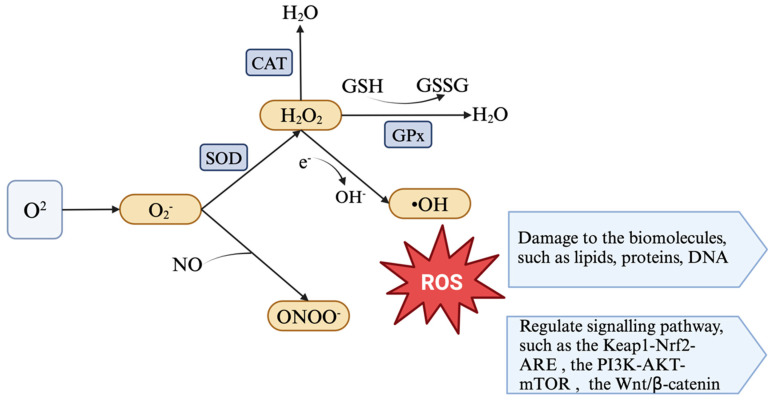
Reactive oxygen species (ROS) are a class of oxygen-containing highly reactive molecules, including superoxide anions (O_2_−), hydrogen peroxide (H_2_O_2_), hydroxyl radicals (−OH), etc. Enzymatic antioxidants are a class of enzymes that catalyse specific reactions to achieve antioxidant effects, including superoxide dismutase (SOD), catalase (CAT), glutathione peroxidase (GPX), etc. The mechanism of ROS injury to the body is mainly manifested in the negative impacts on biomolecules such as lipids, proteins, and DNA. In addition, ROS can also regulate signalling pathways such as the *Keap1-Nrf2-ARE* signalling pathway, the *PI3K-AKT-mTOR* signalling pathway, and the *Wnt/β-catenin* signalling pathway.

For oxidative stress, ROS is one of the significant objects that deserve to be studied. ROS are a class of oxygen-containing highly reactive molecules, including superoxide anions (O_2_−, hydrogen peroxide (H_2_O_2_), hydroxyl radicals (−OH), etc. (Figure 1) [17]. The sources of ROS are diverse. It is reported that ROS can be produced either through normal cellular activities or stimulated by exogenous factors such as radiation [18]. Mitochondria are an important site producing ROS in the cell, mainly through oxidative phosphorylation and the electron transport chain on the inner mitochondrial membrane [19]. Complexes and enzymes involved in mitochondrial respiration, such as complex I, complex III, α-ketoglutarate dehydrogenase (KGDHC), and pyruvate dehydrogenase (PDC), are involved in the production of ROS in mitochondria [20]. In addition to mitochondria, other organelles in the cell, such as peroxisomes and the endoplasmic reticulum (ER) are also sources of ROS [21]. The peroxisome produces ROS such as H_2_O_2_ during metabolism, especially during fatty acid β-oxidation [22].

The endoplasmic reticulum is an important organelle involved in the synthesis and processing of proteins and lipids in the cell and plays an integral role in the *human* body [23,24]. In the ER, protein disulfide isomerase (PDI) introduces disulfide bonds by oxidising sulfhydryl groups in the folded substrate, leaving PDI in a reduced state. The reduced PDI is re-oxidised by endoplasmic reticulum redoxin 1 (*ERO1*), which transfers the gained electrons to molecular oxygen to form H_2_O_2_ [22]. In addition to organelles, ROS can be produced by enzymes such as reduced nicotinamide adenine dinucleotide phosphate oxidase (NADPH oxidase), cytochrome P450, and monoamine oxidase [25,26]. NADPH oxidase is the main source of ROS production in platelets [26]. In addition to the production of ROS from the body, some irritants in vitro may also trigger the production of ROS, such as smoking, radiation, drugs, and certain foods [27]. It has been noted in the literature that tobacco smoke during smoking itself contains ROS, which may lead to oxidative stress, thus damaging DNA, proteins, lipids, and other macromolecules [16]. And it can be deduced that this may be the reason why smoking leads to the formation of malignant tumours. In addition, radiation and some drugs probably also promote a large amount of ROS production. For example, radiotherapy and chemotherapy drugs used commonly and clinically in the treatment of tumours, such as cisplatin, can cause the death of tumour cells by promoting elevated levels of ROS, eventually achieving the goal of hindering the proliferation and metastasis of tumours [19,28]. Notably, iron-rich red meat, trans fatty acids, and large amounts of ethanol possibly promote the formation of ROS [29].

The sources of ROS are rich and varied, and there may be other sources that remain unexplored and need to be investigated in the future.

#### 2.1.2. Definition and Sources of RNS

Like ROS, RNS is another research subject that is closely related to oxidative stress. RNS is a collective term for nitric oxide (NO) and its excitation products in living organisms, which mainly include NO, nitroso anions (ONOO-), nitroxide (NO_2_+), and nitrogen dioxide (NO_2_) [30,31]. The sources of RNS are also diverse. In the *human* body, RNS is mainly produced by nitric oxide synthase catalysing the reaction between L-arginine and oxygen [32]. Moreover, nitrogen in food may be metabolised to reactive nitrogen [30].

RNS, like ROS, plays a very important role in oxidative stress, and its sources are still being explored.

#### 2.1.3. Role of ROS and RNS

It has been found that the effects of ROS and RNS are often closely related to their concentration in the cells and the body. Different levels of ROS and RNS may generally have different effects on cells. In the physiological state, when the concentration of ROS is within the normal range, the cells in the body can maintain the redox balance to promote the successful completion of normal cellular activities [33]. It is found that maintaining normal concentrations of ROS plays a crucial role in transducing cell signals and regulating the expression of genes [34]. However, when the excessive production of ROS and RNS exceeds the antioxidant capacity of the body, oxidative stress occurs, leading to damage to biomolecules. Of course, this may cause the occurrence of a variety of diseases [35]. For malignant tumours, the concentration of ROS and RNS also influences the progression and metastasis of tumour cells [36].When ROS are at a certain level, ROS facilitate the proliferation and migration of cancer cells, thus promoting tumour invasion; however, high concentrations of ROS may lead to the death of cancer cells through cytotoxic effects [17]. Similarly, RNS has a dual role in malignant tumours [37]. Low levels of RNS promote tumour progression by inducing tumour cell proliferation and metastasis, while high levels of RNS cause tumour cell death and inhibit tumour cell metastasis by inducing cytotoxicity [38].

Hence, the roles of ROS and RNS are usually dual, and their concentration often determines tumour progression.

#### 2.1.4. ROS and RNS Damage to the Organism

The mechanism of ROS injury to the body is mainly manifested in the negative impacts on biomolecules such as lipids, proteins, and DNA. RNS is also highly oxidative and can participate in a wide range of biochemical reactions, and excess reactive nitrogen may also cause cellular damage by triggering lipid peroxidation, protein oxidation, and DNA damage [39]. In terms of damaging DNA, ROS or RNS can directly attack nuclear DNA (nDNA) and mitochondrial DNA (mtDNA), resulting in breaks in DNA strands and damage to bases [40,41]. And subsequently, gene mutations and chromosomal instability can be observed. Certainly, the destruction of DNA may easily affect the intracellular signalling pathways and the metabolism of substances, promoting the development of various diseases such as cancer and neurodegenerative diseases [10,19,42]. For tumours, these changes may facilitate the proliferation and invasion of tumour cells and accelerate tumour progression and metastasis.

ROS and RNS attack not only DNA but also proteins, inducing changes in the structure and function of different proteins. Oxidative modifications of proteins are partly reversible, such as the oxidation of cysteine residues, and they are also partly irreversible, such as carbonylation and nitroxylation [43,44]. Irreversible oxidative modifications usually result in the loss of the original function of the protein [16]. Carbonylation is a typically irreversible protein modification, mostly found in histidine and lysine [45]. The products generated by the oxidative modification of proteins may act as signalling molecules to activate signalling pathways associated with tumourigenesis and progression.

In addition to affecting DNA and proteins, ROS and RNS can trigger lipid peroxidation. It is observed that lipid peroxidation can bring about an impaired structure and function of cell membranes and organelles. Obviously, lipids are present in the cell membrane, and the lipid peroxidation induced by ROS is able to destroy the structure and stability of the cell membrane [46]. Furthermore, ROS can induce the degradation of polyunsaturated fatty acids, resulting in the formation of various products such as malondialdehyde (MDA), which may be associated with multiple diseases [42,47]. It has been noted that patients suffering from malignant tumours have more pronounced elevated levels of MDA than normal individuals [48]. Moreover, lipid peroxidation may produce 4-hydroxynonenal (4-HNE) and acrolein, both of which can reduce the antioxidant capacity of cells by binding to glutathione (GSH) in the cells [16,46].

It follows that the destruction of biomolecules is usually the main mechanism by which ROS and RNS lead to cellular damage.

### 2.2. Oxidative Stress in Ovarian Cancer

The presence of oxidative stress in ovarian cancer has been confirmed by high levels of oxidative stress markers in its tissues and cells. The mechanism of oxidative stress generation may involve several aspects. For one thing, if the metabolism of ovarian cancer cells is abnormally active, it may lead to an increase in the production of ROS and RNS, and for another, the antioxidant defence system of ovarian cancer cells may be impaired, which is unable to scavenge the excessive ROS and RNS effectively [49].

#### 2.2.1. Oxidative Stress Promotes Ovarian Cancer

It has been found that oxidative stress contributes to the progression, invasion, and metastasis of ovarian cancer through the following mechanisms:

##### Influencing Genes

It has been found that when ROS levels are maintained in a certain degree of equilibrium with antioxidants, a certain concentration of ROS is maintained in the cells, and this may promote the expression of multidrug resistance genes (e.g., P-glycoprotein (*P-gp*) and multidrug resistance protein 1 (*MRP1*)) in ovarian cancer cells, which ultimately leads to the formation of multidrug resistance [50]. Both *P-gp* and *MRP1* are ATP-binding cassette (ABC) transport proteins, which in cancer usually play a role in multidrug resistance by affecting drug efficacy through the efflux of various types of chemotherapeutic drugs from the cell [51]. Increased *P-gp* has been shown to promote chemotherapeutic drug efflux, leading to the proliferation of ovarian cancer cells resistant to chemotherapeutic drugs such as paclitaxel [52]. Gana et al. suggested that Glutathione (GSH)-depleting *MRP1* modulators may treat chemotherapy-resistant *MRP1* overexpressing ovarian cancers by increasing the concentration of ROS [53].

And it has been found that oxidative stress can also affect genes by oxidatively damaging DNA, affecting DNA repair, and promoting inflammatory responses, thus influencing the occurrence and development of ovarian cancer [54]. One study found that mtDNA mutation due to oxidative damage was associated with the development and progression of epithelial ovarian cancer (EOC) [55]. In a study where female mice were irradiated with iron high charge and energy HZE particles, it was found that iron HZE particles caused ovarian tumourigenesis in mice through oxidative damage [56]. In addition, Mann et al. found that elevated levels of oxidatively damaged DNA may lead to increased expression of programmed cell death ligand 1 (PD-L1) in both mucinous and plasma ovarian cancers. Therefore, PD-L1 inhibitors might be useful for the treatment of ovarian cancer [54]. Additionally, OS can also affect the progression and treatment of ovarian cancer by influencing the expression of microRNAs (miRNAs). MiRNAs, small non-coding RNAs involved in regulating post-transcriptional gene expression, have been found to influence ovarian cancer development by regulating vascular endothelial growth factor A (VEGFA) gene expression [57,58]. In addition, miRNAs influence the proliferation of ovarian cancer cells by participating in the regulation of the inflammatory immune response [59]. Marí-Alexandre et al. indicated that the dysregulation of oxidative stress and miRNA may promote the development of high-grade plasmacytoid ovarian carcinoma (HGSOC) and affect the sensitivity of chemotherapeutic agents for the treatment of HGSOC, which ultimately leads to poor prognosis of patients [60].

It is clear that oxidative stress can affect genes in several ways, thereby promoting the development and metastasis of ovarian cancer.

##### Regulating Signalling Pathway

In addition to affecting genes, oxidative stress can also promote the survival and proliferation of ovarian cancer cells by regulating signalling pathways such as the Kelch-like ECH-associated protein 1 (*Keap1*)–nuclear factor erythroid 2-related factor 2 (*Nrf2*)–antioxidant response element (ARE) signalling pathway, phosphatidylinositol-3-kinase (PI3K)–protein kinase B (AKT)–mammalian target of rapamycin (*mTOR*) signalling pathway, and the *Wnt/β*-catenin signalling pathway.

The *Keap1-Nrf2-ARE* signalling pathway is an important way for the organism to combat oxidative stress, which maintains intracellular redox homeostasis by regulating the transcription of downstream genes, thus exerting anti-inflammatory, anti-cancer, and anti-ageing functions [61,62]. *Nrf2* is a transcription factor that is very sensitive to oxidative stress, and *Keap1*, on the other hand, is present in the cytoplasm as a repressor of *Nrf2* [63]. Normally, *Keap1* binds to *Nrf2* to form a *Keap1-Nrf2* complex [64].

**Figure 2:**
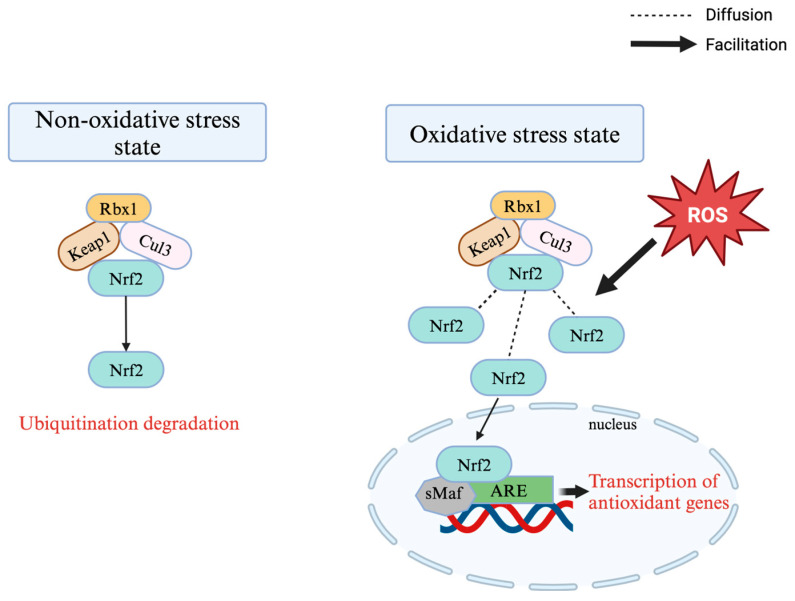
Normally, the Kelch-like ECH-associated protein 1 (*Keap1*) binds to nuclear factor erythroid 2-related factor 2 (*Nrf2*) to form a Keap1-Nrf2 complex. However, under oxidative stress, the conformation of Keap1 changes, resulting in unstable binding to Nrf2. And then Nrf2 separates from Keap1 and is transferred to the nucleus, where it binds to the small muscle tendon membrane fibrosarcoma (Maf) protein and recognises the ARE sequence, which initiates the transcription of the downstream antioxidant genes and exerts antioxidant effects.

However, under oxidative stress, the conformation of *Keap1* changes, resulting in unstable binding to *Nrf2*. And then *Nrf2* separates from *Keap1* and is transferred to the nucleus, where it binds to the small muscle tendon membrane fibrosarcoma (Maf) protein and recognises the ARE sequence, which initiates the transcription of the downstream antioxidant genes and exerts antioxidant effects [65] (Figure 2).

It was found that under oxidative stress, the *Keap1-Nrf2-ARE* pathway could not only reduce the damage of oxidative stress on ovarian cancer cells by inducing the production of antioxidant enzymes, such as glutathione peroxidase (GPX), but also help tumour cells to resist the attack of chemotherapeutic drugs and enhance the chemoresistance of ovarian cancer cells by activating the expression of downstream detoxification genes [66]. By analysing the data, Xia et al. demonstrated that cisplatin resistance in *human* ovarian cancer cells was attributed to the regulation of the *Keap1-Nrf2-ARE* signalling pathway by ovarian cancer cells through the high expression of p62 [67]. Another study showed that the overexpression of *p62* may protect cells from oxidative damage by activating the *Keap1-Nrf2-ARE* signalling pathway in ovarian cancer [68]. Therefore, the *Keap1-Nrf2-ARE* pathway may be a potential target in ovarian cancer therapy, and by inhibiting *Nrf2* activity or interfering with its signalling pathway, the efficacy of chemotherapeutic agents may be improved and the resistance of tumour cells reversed [66]. However, it should be noted that the *Nrf2* pathway may also inhibit ovarian carcinogenesis [69].

The *PI3K-AKT-mTOR* signalling pathway is an important intracellular signalling pathway that is involved in the regulation of several aspects of cell growth, proliferation, apoptosis, and metabolism [70,71]. Aberrant activation of the *PI3K-AKT-mTOR* signalling pathway is often observed in ovarian cancer [72]. This aberrant activation may be caused by ROS [73]. The aberrant activation of the *PI3K-AKT-mTOR* signalling pathway by ROS promotes the growth, proliferation, and invasive ability of ovarian cancer cells while inhibiting apoptosis. This may accelerate ovarian cancer progression and lead to drug resistance and poor prognosis [74,75].

The *Wnt/β*-catenin signalling pathway plays an important role in ovarian cancer development and progression [76]. The *Wnt/β*-catenin signalling pathway includes the classical *Wnt* pathway as well as the non-classical *Wnt* pathway that does not depend on β-catenin transcriptional activity [77]. Abnormal activation of this pathway affects the invasive and migratory abilities of ovarian cancer cells and reduces the sensitivity of ovarian cancer cells to chemotherapeutic agents [78]. For example, Ji et al. found that Forkhead Box P4 (FOXP4) can activate the *Wnt* signalling pathway by inducing the expression of protein tyrosine kinase 7, thereby promoting the development of ovarian cancer [79]. In addition, it was noted that ubiquitin-specific peptidase 43 (USP43) could reduce the sensitivity of epithelial ovarian cancer cells to cisplatin by activating the Wnt/β-catenin signalling pathway [80]. Apparently, oxidative stress can also activate the Wnt/β-catenin signalling pathway [81]. Therefore, in ovarian cancer, oxidative stress can promote the proliferation and metastasis of ovarian cancer cells and increase the chemoresistance of ovarian cancer cells through the aberrant activation of the *Wnt/β*-catenin signalling pathway, which drives the progression of ovarian cancer. In addition, activation of the Notch pathway has been found to promote ovarian cancer progression [82]. For example, Diao et al. showed that laminin α5 (LAMA5) induced ovarian cancer progression by activating the Notch pathway [83]. The Notch pathway has also been shown to affect the growth and metastasis of ovarian cancer cells by influencing the infiltration of immune cells. In addition, Bocchicchio et al. showed that the Notch pathway can synergise with the Wnt/β-catenin signalling pathway to promote the proliferation and metastasis of ovarian cancer cells [84].

The regulation of signalling pathways is also one of the important ways in which ovarian cancer progression is affected, and although some of these signalling pathways have been explored, there are other relevant signalling pathways that need to continue to be explored.

##### Modifying the Tumour Microenvironment

Studies have shown that oxidative stress can also influence ovarian cancer progression by altering the tumour microenvironment.

The tumour microenvironment (TME) is composed of the tumour cells, the surrounding blood vessels, immune cells, fibroblasts, the extracellular matrix, and many other components, which interact with each other and collectively influence tumour growth, progression, and metastasis [85]. ROS not only induces angiogenesis to promote the proliferation and migration of tumour cells but also facilitates immune escape and accelerates the progression of ovarian cancer by affecting a variety of immune cells in the tumour microenvironment [19,86].

Tumour-associated macrophages (TAMs) are a numerous and important class of cells in the TME, which are divided into M1-like macrophages and M2-like macrophages, and they are closely related to the progression of ovarian cancer [87]. Oxidative stress not only promotes TAMs to secrete a variety of growth factors, such as VEGF and transforming growth factor-β (TGF-β), to promote the proliferation and angiogenesis of ovarian cancer cells but also promotes the secretion of chemokines by TAMs to promote the migration and invasive ability of ovarian cancer cells, which increases their metastatic risk [88,89]. Oxidative stress in the ovarian cancer microenvironment can induce the expression of M2-like macrophages [90]. The M2-like TAMs can inhibit the activation and function of T cells through the secretion of immunosuppressive cytokines, such as IL-10 and TGF-β, which can help ovarian cancer cells evade the attack of the immune system [91]. In addition to TAMs, other immune cells, such as T regulatory cells (Treg cells) and myeloid-derived suppressor cells (MDSCs), also play an important role in the development of ovarian cancer. Oxidative stress can shape the peritoneal TME by recruiting and regulating MDSCs and Treg cells, creating a permissive environment for tumour development, and promoting the spread and metastasis of tumour cells [92].

In addition to cells, ascites is an important mediator in the ovarian cancer tumour microenvironment, which can help tumour cells spread to pelvic organs and even extra-pelvic organs [93]. Oxidative stress promotes the spread of ovarian cancer cells through ascites, which ultimately favours the spread and metastasis of ovarian cancer [94].

In conclusion, oxidative stress promotes ovarian cancer proliferation and invasion in a variety of ways, including altering genes, regulating signalling pathways, and altering the tumour microenvironment, and there may be other ways that deserve continued exploration and research.

#### 2.2.2. Treating Ovarian Cancer by Oxidative Stress

It is worth noting that although oxidative stress in the body promotes the development of ovarian cancer, chemotherapeutic drugs, radiotherapy, and other treatment modalities are used to induce the death of ovarian cancer cells by increasing the concentration of ROS, which ultimately achieves the goal of treatment [19]. For example, cisplatin, one of the effective and widely used chemotherapeutic agents for the treatment of ovarian cancer, causes ovarian cancer cell death by inducing the mitochondria-dependent ROS to damage nuclear DNA [17]. It is well known that poly ADP-ribose polymerase (PARP) has the function of sensing and repairing DNA strand breaks, and PARP inhibitors inhibit ovarian cancer development by inhibiting DNA repair in ovarian cancer cells; in addition to this, PARP inhibitors can promote oxidative stress in ovarian cancer cells to achieve anti-tumour effects [95]. In addition, Kanakkanthara et al. noted that the combination of ceritinib and PARP inhibitors enhanced the inhibitory effect of PARP inhibitors on ovarian cancer by taking advantage of the fact that ceritinib, an anaplastic lymphoma kinase (ALK) inhibitor, can induce oxidative stress and promote oxidative DNA damage by increasing ROS [96]. In one study, the synthetic β-nitrostyrene derivative CYT-Rx20 was synthesised, which can induce apoptosis and inhibit the development of ovarian cancer cells by promoting the generation of ROS and leading to DNA damage [97].Therefore, CYT-Rx20 may be useful for the treatment of ovarian cancer but studies are needed to confirm this.

Furthermore, some previous studies have explored some natural or synthetic substances to treat ovarian cancer by inducing oxidative stress. For example, berberine, a compound present in many herbal plants, promotes oxidative DNA damage and down-regulates homologous recombination repair in ovarian cancer cells [98]. Hou et al. demonstrated that berberine sensitised cancer cells to PARP inhibition and that the combined use of berberine and a PARP inhibitor significantly promoted apoptosis and inhibited tumour growth [98]. Aleissa et al. noted that the use of berberine promoted oxidative damage and inhibited the proliferation of ovarian cancer cells induced by radiotherapy [99]. Sahai et al. noted that gedunin isolated from plants promotes the production of large amounts of ROS, leading to oxidative DNA damage, cell cycle arrest, and thus the inhibition of cell proliferation [100]. So, gedunin may be useful for the treatment of ovarian cancer but research is needed to support this. Furthermore, a study found that the extract of vernonia calvoana (VC), a valuable plant, can induce DNA damage and cell cycle arrest in ovarian cells through oxidative stress, thus promoting apoptosis and inhibiting the proliferation of ovarian cancer cells [101].

In addition, hyperthermia therapy (HT) increases the efficacy of PARP inhibitors in the treatment of ovarian cancer, and the combination of HT with PARP inhibitors, such as olaparib, promotes oxidative stress and exacerbates oxidative DNA damage [102].

Different levels of oxidative stress may have opposite effects on ovarian cancer and therefore, oxidative stress is a key focus in the exploration of ovarian cancer treatments. More research is needed to analyse the mechanisms by which oxidative stress affects ovarian cancer and to develop new therapeutic modalities based on it.

## 3. Role of Antioxidants in Ovarian Cancer

### 3.1. Definition, Classification, and Function of Antioxidants

In order to prevent the excessive production of ROS, cells in the body eliminate ROS through antioxidant defence mechanisms, such as endogenous or exogenous antioxidants [103]. Antioxidants are compounds that can scavenge ROS, prevent oxidative damage, and maintain redox balance [104]. Enzymatic antioxidants are a class of enzymes that catalyse specific reactions to achieve antioxidant effects, including superoxide dismutase (SOD), catalase (CAT), and GPX [105] (Figure 1). SOD, which is a class of enzymes that catalyse the conversion of O_2_− to H_2_O_2_ and O_2_, can be found in a wide range of organisms including bacteria, fungi, plants, and animals [106]. CAT is responsible for the breakdown of hydrogen peroxide into water and oxygen, thereby removing hydrogen peroxide radicals in the body [46,105]. GPX catalyses the reduction reaction of GSH with hydrogen peroxide or other peroxides to produce oxidised glutathione (GSSG) and water under the condition that GSH acts as a substrate, ultimately reducing peroxide radicals in the body [19,107].

Non-enzymatic antioxidants are compounds that directly remove free radicals or inhibit oxidative reactions, including vitamin C, vitamin E, melatonin, carotenoids, flavonoids, and GSH [108,109]. Vitamin C, vitamin E, carotenoids, and flavonoids are antioxidants that can enter the body through exogenous routes such as diet [35]. Among them, vitamin C is a class of water-soluble vitamins that scavenges free radicals and exerts antioxidant effects in the body [110]. Vitamin E (α-tocopherol) is a fat-soluble vitamin with antioxidant effects that can prevent cell membranes from being attacked by ROS [29]. GSH is an abundant endogenous antioxidant that is synthesised from glutamate, cysteine, and glycine in a two-step process catalysed by various enzymes [111,112]. GSH regulates redox homeostasis by participating in the reduction of H_2_O_2_ by glutathione peroxidases (GPXs) and glutathione transferases (GSTs) [113].

Suppose ROS in the body is excessive and leads to oxidative stress. In that case, antioxidants will exert their protective effects through various mechanisms, including directly clearing ROS, repairing oxidative damage, and up-regulating endogenous antioxidant defences. On the one hand, antioxidants can directly react with ROS and reduce the concentration of ROS, thus alleviating the cellular damage caused by oxidative stress, and on the other hand, certain antioxidants can up-regulate the expression of antioxidant enzymes (e.g., SOD, CAT, etc.) and enhance the antioxidant capacity of cells [106,114].

### 3.2. Role of Antioxidants or Regulators of Antioxidants in Ovarian Cancer

Antioxidants are a class of compounds that scavenge ROS and RNS or inhibit their production and have the ability to protect cells from oxidative damage [14]. The role of antioxidants in malignant tumours is dual. Antioxidants have potential anti-tumour effects but may also promote tumour growth and metastasis in some cases [111]. Alternatively, certain regulators of antioxidants can indirectly affect tumour proliferation and invasion by modulating the production of antioxidants.

#### 3.2.1. Anti-Tumour Effects

Some antioxidants have shown significant anti-tumour effects in ovarian cancer. Lycopene, a kind of carotenoid, was found to exert a powerful antioxidant capacity by eliminating free radicals [115]. Holzapfel et al. stated that the prophylactic use of lycopene significantly reduced the metastatic load in mice carrying ovarian cancer, whereas treating mice carrying ovarian cancer with lycopene saw a significantly reduced tumour load [116]. What is more, this study also observed that lycopene not only positively promotes the inhibitory effects of paclitaxel and carboplatin on tumour cells but also reduces the expression of cancer antigen 125 (CA125) [116]. This shows that lycopene can synergise with chemotherapeutic agents to inhibit the progression and metastasis of ovarian cancer, but further studies are needed to confirm this conclusion. Other antioxidants such as vitamin C have shown anti-tumour effects in ovarian cancer by inhibiting the proliferation, invasion, and metastasis of ovarian cancer cells [117,118,119]. In a prospective cohort study, it was noted that a higher intake of vitamin C and β-carotene prior to diagnosis improved the survival rate of patients with ovarian cancer [120]. Rosmarinic acid, a natural antioxidant, can inhibit oxidative stress, promote the production of antioxidants, and help to inhibit the progression of ovarian cancer [121]. However, the anti-tumour mechanisms of these antioxidants may involve a variety of biological processes, such as inhibiting cell proliferation, inducing apoptosis, and regulating redox balance. But the specific mechanisms have not yet been fully elucidated. Therefore, appropriate antioxidants for the treatment of ovarian cancer should be selected after the anti-tumour mechanisms of antioxidants are fully understood and the specific condition of the patient is fully considered.

#### 3.2.2. Promoting Tumour Proliferation and Metastasis

**Figure 3:**
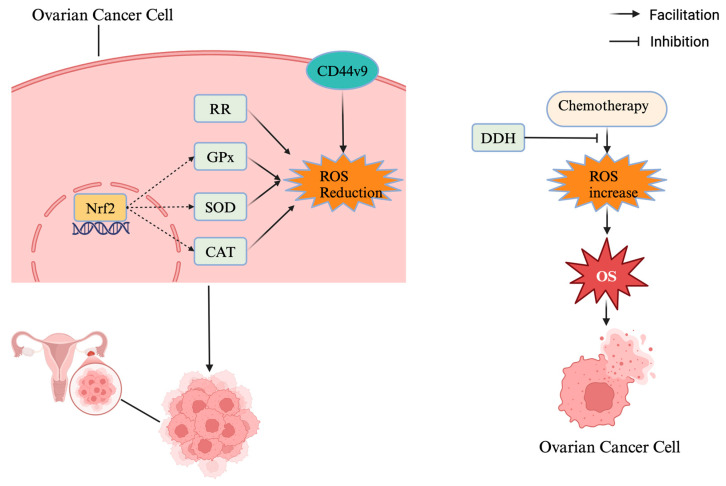
In ovarian cancer, the activation of nuclear factor erythroid 2-related factor 2 (Nrf2) promotes the expression of antioxidant enzymes, such as catalase (CAT), glutathione peroxidase (GPx), and superoxide dismutase (SOD), which enhance the antioxidant capacity of ovarian cancer cells. Thioredoxin reductase (RR), an antioxidant enzyme, also promotes the development of ovarian cancer. CD44v9 protects ovarian cancer cells from oxidative stress damage by reducing ROS levels. Increased dihydrodiol dehydrogenase (DDH) activity may reduce the cisplatin-induced production of free radicals, which reduces the efficacy of cisplatin in the treatment of ovarian cancer.

While some antioxidants exhibit anti-tumour effects in ovarian cancer, others or their modulators may stimulate cancer growth and metastasis (Figure 3). It has been found that for cancer patients and those at elevated risk of cancer, the additional intake of antioxidants may stimulate tumour angiogenesis and promote the growth and spread of cancer cells [114]. Some antioxidants such as N-acetylcysteine (NAC), despite exhibiting antioxidant and anti-tumour effects in some studies, have been found to potentially promote the growth and metastasis of ovarian cancer in other studies [122]. For example, NAC may promote the proliferation of ovarian cancer cells by hindering the anti-tumour effects of *PARP* inhibitors [123]. In addition, resveratrol has been found to cause the death of ovarian cancer cells, but NAC can promote ovarian cancer progression by inhibiting the function of resveratrol [124].

GPX is a family of enzymes that regulates redox homeostasis and reduce cellular damage caused by oxidative stress [125]. Glutathione peroxidase 3 (GPX3), as a member of the GPX family, can scavenge free radicals such as hydrogen peroxide for achieving the goal of protecting cells. Some studies have shown that a high expression of GPX3 can inhibit the development of malignant tumours such as gastric cancer and endometrial cancer, but for ovarian cancer, on the contrary, a high expression of GPX3 promotes the progression and recurrence of ovarian cancer and reduces the survival rate of patients [126,127].

Superoxide dismutase 2 (SOD2), an antioxidant enzyme in mitochondria, scavenges free radicals in the body and reduces oxidative damage to cells caused by free radicals. It has been reported that SOD2 is closely related to the proliferation and metastasis of ovarian clear cell carcinoma. SOD2 may promote the progression of ovarian cancer; thus, it is suggested that inhibiting the expression of SOD2 may hinder the development and invasion of ovarian cancer by increasing the level of ROS that oxidatively damages ovarian cancer cells [128].

Thioredoxin reductase (RR), another antioxidant enzyme involved in reduction reactions in vivo, has also been implicated in the development of ovarian cancer, and studies have confirmed that inhibitors of RR, such as auranofin (AF), can be used to treat ovarian cancer by depleting GSH and promoting oxidative stress [129].

Ovarian cancer cells lacking BRCA1 have been reported to be more sensitive to AF therapy [130]. Typically, the BRCA1 gene acts as an oncogene that repairs DNA damage, promotes the expression of antioxidant genes, and protects cells from oxidative stress [131]. However, it has been suggested that the BRCA1 gene can also reduce the damage to ovarian cancer cells by activating the antioxidant defence system, whereas the BRCA gene deletion disrupts the redox balance by affecting the expression of Nrf2, leading to the excessive production of ROS and, ultimately, to the formation of oxidative stress that damages ovarian cancer cells [132].

*Nrf2*, a nuclear transcription factor, plays an important role as a regulator of antioxidants in modulating the antioxidant capacity of cells and resisting oxidative stress damage [133]. For one thing, in the state of oxidative stress, *Nrf2* activates and enters the nucleus, binds to AREs, and promotes the expression of antioxidant genes, thereby inhibiting oxidative damage in the cell [134]. For another, the activation of the *Nrf2* pathway by oxidative stress promotes the expression of antioxidant enzymes, such as catalase, glutathione peroxidase, and superoxide dismutase, which enhances the antioxidant capacity of the cells and reduces oxidative damage [135]. However, it is found that in ovarian cancer, the activation of *Nrf2* may increase the antioxidant capacity and drug resistance of ovarian cancer cells [136]. The *Nrf2* pathway promotes the proliferation of ovarian cancer cells and the generation of chemoresistance by inhibiting oxidative stress induced by chemotherapeutic drugs, ultimately promoting the progression and metastasis of ovarian cancer [69].

In addition to *Nrf2*, CD44 variant isoform 9 (CD44v9) can also influence ovarian cancer development by regulating redox homeostasis. CD44v9 in ovarian cancer cells protects ovarian cancer cells from oxidative stress by reducing ROS levels [137]. It has been found that in order to achieve antioxidants, ovarian cancer cells up-regulate CD44v9 [138]. Apparently, CD44v9 can promote the proliferation and metastasis of ovarian cancer cells, from which it can be inferred that inhibition of CD44v9 can promote the death of ovarian cancer cells, thus achieving the anti-tumour purpose. Kobayashi et al. showed that the simultaneous inhibition of CD44v9 and Nrf2 may elevate ROS levels, thereby inhibiting tumour progression and increasing the efficacy of chemotherapy through oxidative stress [132].

Dihydrodiol dehydrogenase (DDH) is a member of the aldehyde-keto reductase family, and its role as an antioxidant may also promote the proliferation of ovarian cancer cells [139]. Some studies have proved that the expression of DDH may affect the sensitivity of ovarian cancer to platinum drugs, which in turn may influence the treatment of ovarian cancer and promote the development and metastasis of ovarian cancer. Deng et al. analysed cisplatin-sensitive and cisplatin-resistant ovarian cancer cells in *humans* by using cDNA microarrays and found that the gene for DDH was expressed in cisplatin-resistant ovarian cancer cells more [140]. In addition, Chen et al. demonstrated the increased expression of DDH in *human* ovarian cancer [139]. Additionally, it is found that increased DDH may reduce the cisplatin-induced production of free radicals, which could reduce the effectiveness of cisplatin in treating ovarian cancer and lead to cisplatin resistance [141]. For example, one study demonstrated that an increased expression of DDH may cause a decrease in the production of ROS, which reduces platinum-induced oxidative stress damage to ovarian cancer cells and ultimately promotes the development of cisplatin resistance in ovarian cancer cells [142].

Based on the available research, some antioxidants have been shown to exert anti-tumour effects and some may promote tumour growth and metastasis. More research is needed in the future to confirm the role of the identified antioxidants and to explore the possible role of new antioxidants.

## 4. Antioxidants for Treating Ovarian Cancer

Despite the dual role of antioxidants in ovarian cancer, the strategy of treating ovarian cancer with antioxidants is still of high value and has promising applications. In recent years, the use of many types of antioxidants, such as vitamin-based antioxidants and hormone-based antioxidants, has been widely studied for the treatment of ovarian cancer.

Some antioxidants such as vitamin C, vitamin E, carotenoids, and selenium might be used in the treatment of ovarian cancer. As mentioned earlier, lycopene, a type of carotenoid, can be used to treat ovarian cancer by inhibiting its progression [116]. Additionally, Choi et al. noted that high doses of selenium could inhibit the proliferation of ovarian cancer cells by reducing the levels of GPX3 [143]. However, due to the dual role of these antioxidants in ovarian cancer, the dose and route of administration need to be carefully selected when using them to avoid producing the opposite effect.

Green tea polyphenols, also known as catechins, are one of the components of green tea [144]. Epigallocatechin gallate (EGCG), a derivative of green tea polyphenols, has been gradually coming into the public eye and has been investigated for its anti-inflammatory, antioxidant, and anti-tumour properties [145]. EGCG may be able to treat ovarian cancer by inhibiting the signalling pathways associated with tumourigenesis, but further studies are still needed to confirm this theory [146].

N-acetyl-5-methoxytryptamine (melatonin), as a hormonal antioxidant, may be a new strategy for the treatment of ovarian cancer [147]. Melatonin, also known as pineal gland hormone, is an indole amine that is widely found in a variety of organisms [148]. It has been found that the main site of the body that synthesises and secretes melatonin is the pineal gland but of course, other organs, such as the retina and the gastrointestinal tract, can also produce melatonin [149].

Melatonin has a variety of important physiological functions in the body; notably, melatonin, as an antioxidant, has a powerful antioxidant capacity. On the one hand, melatonin can directly eliminate free radicals in the body and reduce cellular damage caused by oxidative stress; on the other hand, melatonin can also enhance the activity of antioxidant enzymes and protect biological macromolecules, such as proteins, lipids, and DNA, from oxidative damage caused by free radicals [150]. In recent years, more and more attention has been paid to the value of melatonin in the treatment of cancer, mood disorders, sleep disorders, and other diseases. So far, a number of studies have found that melatonin can inhibit the proliferation and migration of ovarian cancer cells through multiple pathways. Moreover, melatonin can also promote the apoptosis of ovarian cancer cells [151]. Surprisingly, melatonin also protects the ovaries during the use of chemotherapeutic drugs and reduces the effects of chemotherapeutic drugs on fertility [152]. Of course, side effects can occur when the level of melatonin is too high, such as dizziness and headaches, nausea, drowsiness, and loss of vision [152]. Hence, more studies are needed in the future to fully analyse the anti-tumour mechanism and safety of melatonin.

## 5. Antioxidants for the Prevention of Ovarian Cancer

In addition to being used in the treatment of ovarian cancer, antioxidants may also be considered as a potential strategy for the prevention of ovarian cancer. A large body of previous research has analysed the relationship between the intake of antioxidant-rich foods or antioxidant supplementation and the risk of developing ovarian cancer. One study suggested that the supplementation of selenium may somewhat reduce the risk of ovarian cancer in African American women [153]. Moreover, some antioxidants such as resveratrol and curcumin have been suggested to have a preventive effect against ovarian cancer.

Resveratrol is a natural compound that performs several functions in living organisms, such as resisting oxidation, inhibiting inflammatory responses, and hindering angiogenesis [154]. Resveratrol has been identified to be used in the prevention and treatment of several cancers, ovarian cancer being one of them [155]. For one thing, resveratrol has antioxidant and anti-inflammatory effects to reduce the damage to the ovaries caused by oxidative stress and inflammatory responses, and on the other hand, resveratrol can also promote the immune system in the body to perform anti-tumour immune functions [156]. Therefore, it can be concluded that resveratrol can reduce the risk of ovarian cancer and can be used for the prevention and treatment of ovarian cancer.

Curcumin is a natural substance derived from turmeric rhizomes that exerts powerful anti-tumour, antioxidant, anti-inflammatory, and anti-angiogenic effects [157]. Studies have shown that by regulating redox balance, curcumin can not only inhibit the progression of ovarian cancer but also assist in increasing the efficacy of chemotherapeutic drugs by reversing the resistance of ovarian cancer cells to chemotherapeutic drugs such as cisplatin [158]. Therefore, curcumin may also play an important role in the prevention and treatment of ovarian cancer.

Additionally, some antioxidants targeting ROS may be useful for preventing cancers due to the oncogenic effects of ROS in some cases, but the specific role of such antioxidants in ovarian cancer needs to be further verified [159]. What is more, the preventive role of vitamin C, also known as ascorbic acid, which can be obtained from fruits and vegetables, in ovarian cancer has not been clearly established so far. Some studies have concluded that vitamin C prevents the development of ovarian cancer, but others have pointed out that a higher intake of vitamin C from diet increases the risk of ovarian cancer [160].

It is important to focus on the fact that there is still some controversy and uncertainty about the role of antioxidants in the prevention of ovarian cancer. It has been shown that the intake of antioxidants does not significantly reduce the risk of ovarian cancer. For example, Koushik et al. pointed out that the intake of vitamins A, C, and E was not significantly associated with the risk of ovarian cancer [161]. Also, Long et al. noted that taking vitamin C from the diet was not related to the risk of ovarian cancer [162]. Of note, in a large study evaluating the relationship between the intake of vitamins A, C, and E and specific carotenoids and the risk of ovarian cancer in 80,326 women, no significant association was found between the exogenous intake of these antioxidants and the risk of ovarian cancer [163]. Therefore, using antioxidants to prevent the occurrence of ovarian cancer needs to be carefully considered and selected on an individual basis to avoid counterproductive phenomena.

## 6. Conclusions and Discussion

Ovarian cancer is one of the difficult problems in gynaecology, which seriously threatens women’s health and lives due to its poor treatment outcome and high recurrence rate. The mechanism of ovarian cancer development and its treatment modalities have been constantly explored. Although ovarian cancer is now better understood than before and there are specific treatment protocols in clinical practice, it is still necessary to continue in-depth research and dedicate ourselves to finding more and better treatments in order to improve therapeutic efficacy, prolong the survival period of the patients, and increase the survival rate.

Oxidative stress is a state of imbalance between the production of ROS and RNS and the antioxidant defence system in the organism. With a deeper understanding of oxidative stress, it has been found that oxidative stress plays a key role in the development, progression, and treatment resistance of many diseases such as ovarian cancer. ROS plays a very crucial role in oxidative stress. Numerous studies have confirmed that the effects of ROS on both normal and cancer cells are dual. For normal cells, a certain range of ROS is beneficial to maintain normal cellular activities, but a high level of ROS may trigger oxidative stress and lead to the development of various diseases. For cancer cells, a certain range of ROS promotes the proliferation and metastasis of cancer cells and facilitates tumour development; however, a high level of ROS can lead to the death of cancer cells. Currently, the conventional treatment protocol for ovarian cancer, chemotherapy, fully applies the killing effect of a high concentration of ROS on ovarian cancer cells. Notably, different concentrations of ROS have different effects on a variety of immune cells in the tumour microenvironment. A certain range of ROS can promote immune cells to give full play to anti-tumour immune functions, but a high concentration of ROS may inhibit normal anti-tumour immune responses and promote tumour progression [28]. Therefore, it is necessary to continue to analyse the duality of ROS in depth in the future and explore new strategies for the treatment of ovarian cancer on this basis.

Antioxidants cannot be ignored as oxidative stress markers in the prevention and treatment of ovarian cancer. Some antioxidants may attenuate oxidative damage induced by oxidative stress, inhibit tumour cell growth, and provide promising therapeutic strategies for patients diagnosed with ovarian cancer, while some other antioxidants may promote the survival, proliferation, and metastasis of ovarian cancer cells. Therefore, selecting appropriate and effective antioxidants is crucial for the treatment of ovarian cancer. Additionally, not enough research has been performed on antioxidants to understand the specific mechanism of antioxidants in ovarian cancer. In the future, more studies are needed to explore the specific processes by which antioxidants inhibit the proliferation, apoptosis, and metastasis of ovarian cancer cells. It is also necessary to assess the safety and side effects of antioxidants in ovarian cancer treatment, especially the risks and potential harms of the long-term use of antioxidants. The mechanisms, effects, and selection of preventive regimens of antioxidants for ovarian cancer prevention are equally important. Notably, because of the many types of ovarian cancer and individual differences between patients, unique protocols should be developed when choosing antioxidants. Moreover, a combination of antioxidants and chemotherapeutic agents can be considered for the treatment of ovarian cancer in order to improve the efficacy and safety of the treatment and reduce the incidence of adverse effects.

In the future, a further in-depth exploration of the interaction mechanisms between oxidative stress and ovarian cancer, as well as the role of antioxidants in ovarian cancer, is needed to provide new ideas and approaches for the prevention and treatment of ovarian cancer.

## References

[1] Webb P.M., Jordan S.J. (2024). Global epidemiology of epithelial ovarian cancer. Nat. Rev. Clin. Oncol..

[2] Tian W., Lei N., Zhou J., Chen M., Guo R., Qin B., Li Y., Chang L. (2022). Extracellular vesicles in ovarian cancer chemoresistance, metastasis, and immune evasion. Cell Death Dis..

[3] Schoutrop E., Moyano-Galceran L., Lheureux S., Mattsson J., Lehti K., Dahlstrand H., Magalhaes I. (2022). Molecular, cellular and systemic aspects of epithelial ovarian cancer and its tumor microenvironment. Semin. Cancer Biol..

[4] Amano T., Murakami A., Murakami T., Chano T. (2021). Antioxidants and Therapeutic Targets in Ovarian Clear Cell Carcinoma. Antioxidants.

[5] Zhang R., Siu M.K.Y., Ngan H.Y.S., Chan K.K.L. (2022). Molecular Biomarkers for the Early Detection of Ovarian Cancer. Int. J. Mol. Sci..

[6] Cabasag C.J., Fagan P.J., Ferlay J., Vignat J., Laversanne M., Liu L., van der Aa M.A., Bray F., Soerjomataram I. (2022). Ovarian cancer today and tomorrow: A global assessment by world region and Human Development Index using GLOBOCAN 2020. Int. J. Cancer.

[7] Konstantinopoulos P.A., Matulonis U.A. (2023). Clinical and translational advances in ovarian cancer therapy. Nat. Cancer.

[8] Jiang H., Zuo J., Li B., Chen R., Luo K., Xiang X., Lu S., Huang C., Liu L., Tang J. (2023). Drug-induced oxidative stress in cancer treatments: Angel or devil?. Redox Biol..

[9] Xiao H.H. (2024). The Role of Oxidative Stress and Natural Products in Maintaining Human Health. Nutrients.

[10] Klaunig J.E. (2018). Oxidative Stress and Cancer. Curr. Pharm. Des..

[11] Li K., Deng Z., Lei C., Ding X., Li J., Wang C. (2024). The Role of Oxidative Stress in Tumorigenesis and Progression. Cells.

[12] Stieg D.C., Wang Y., Liu L.-Z., Jiang B.-H. (2022). ROS and miRNA Dysregulation in Ovarian Cancer Development, Angiogenesis and Therapeutic Resistance. Int. J. Mol. Sci..

[13] Pei H., Yang Y., Cui L., Yang J., Li X., Yang Y., Duan H. (2016). Bisdemethoxycurcumin inhibits ovarian cancer via reducing oxidative stress mediated MMPs expressions. Sci. Rep..

[14] Hecht F., Zocchi M., Alimohammadi F., Harris I.S. (2024). Regulation of antioxidants in cancer. Mol. Cell.

[15] Dong C., Zhang N.J., Zhang L.J. (2021). Oxidative stress in leukemia and antioxidant treatment. Chin. Med. J. Engl..

[16] Caliri A.W., Tommasi S., Besaratinia A. (2021). Relationships among smoking, oxidative stress, inflammation, macromolecular damage, and cancer. Mutat. Res. Rev. Mutat. Res..

[17] Nakamura H., Takada K. (2021). Reactive oxygen species in cancer: Current findings and future directions. Cancer Sci..

[18] Wang Y., Qi H., Liu Y., Duan C., Liu X., Xia T., Chen D., Piao H.L., Liu H.X. (2021). The double-edged roles of ROS in cancer prevention and therapy. Theranostics.

[19] Kuo C.L., Ponneri Babuharisankar A., Lin Y.C., Lien H.W., Lo Y.K., Chou H.Y., Tangeda V., Cheng L.C., Cheng A.N., Lee A.Y. (2022). Mitochondrial oxidative stress in the tumor microenvironment and cancer immunoescape: Foe or friend?. J. Biomed. Sci..

[20] Zorov D.B., Juhaszova M., Sollott S.J. (2014). Mitochondrial reactive oxygen species (ROS) and ROS-induced ROS release. Physiol. Rev..

[21] Lin Y., Jiang M., Chen W., Zhao T., Wei Y. (2019). Cancer and ER stress: Mutual crosstalk between autophagy, oxidative stress and inflammatory response. Biomed. Pharmacother..

[22] Forrester S.J., Kikuchi D.S., Hernandes M.S., Xu Q., Griendling K.K. (2018). Reactive Oxygen Species in Metabolic and Inflammatory Signaling. Circ. Res..

[23] Iurlaro R., Munoz-Pinedo C. (2016). Cell death induced by endoplasmic reticulum stress. FEBS J..

[24] Quintanilha J.C.F. (2017). Involvement of cytochrome P450 in cisplatin treatment: Implications for toxicity. Cancer Chemother. Pharmacol..

[25] Cheung E.C. (2022). The role of ROS in tumour development and progression. Nat. Rev. Cancer.

[26] Fuentes E. (2018). NADPH oxidase 2 (NOX2): A key target of oxidative stress-mediated platelet activation and thrombosis. Trends Cardiovasc. Med..

[27] Sies H., Jones D.P. (2020). Reactive oxygen species (ROS) as pleiotropic physiological signalling agents. Nat. Rev. Mol. Cell Biol..

[28] Zheng Z., Su J., Bao X., Wang H., Bian C., Zhao Q., Jiang X. (2023). Mechanisms and applications of radiation-induced oxidative stress in regulating cancer immunotherapy. Front. Immunol..

[29] Jomova K., Raptova R., Alomar S.Y., Alwasel S.H., Nepovimova E., Kuca K., Valko M. (2023). Reactive oxygen species, toxicity, oxidative stress, and antioxidants: Chronic diseases and aging. Arch. Toxicol..

[30] Radi R. (2018). Oxygen radicals, nitric oxide, and peroxynitrite: Redox pathways in molecular medicine. Proc. Natl. Acad. Sci. USA.

[31] Adams L., Franco M.C., Estevez A.G. (2015). Reactive nitrogen species in cellular signaling. Exp. Biol. Med..

[32] Al-Shehri S.S. (2021). Reactive oxygen and nitrogen species and innate immune response. Biochimie.

[33] Glasauer A., Chandel N.S. (2014). Targeting antioxidants for cancer therapy. Biochem. Pharmacol..

[34] Wu S., Jiang L., Lei L., Fu C., Huang J., Hu Y., Dong Y., Chen J., Zeng Q. (2023). Crosstalk between G-quadruplex and ROS. Cell Death Dis..

[35] Jomova K., Alomar S.Y., Alwasel S.H., Nepovimova E., Kuca K., Valko M. (2024). Several lines of antioxidant defense against oxidative stress: Antioxidant enzymes, nanomaterials with multiple enzyme-mimicking activities, and low-molecular-weight antioxidants. Arch. Toxicol..

[36] Hayes J.D., Dinkova-Kostova A.T., Tew K.D. (2020). Oxidative Stress in Cancer. Cancer Cell.

[37] Sanhueza C., Araos J., Naranjo L., Barros E., Subiabre M., Toledo F., Gutiérrez J., Chiarello D.I., Pardo F., Leiva A. (2016). Nitric oxide and pH modulation in gynaecological cancer. J. Cell. Mol. Med..

[38] El-Sehemy A., Postovit L.M., Fu Y. (2016). Nitric oxide signaling in human ovarian cancer: A potential therapeutic target. Nitric Oxide.

[39] Lushchak V.I., Lushchak O. (2021). Interplay between reactive oxygen and nitrogen species in living organisms. Chem. Biol. Interact..

[40] Ionescu-Tucker A., Cotman C.W. (2021). Emerging roles of oxidative stress in brain aging and Alzheimer’s disease. Neurobiol. Aging.

[41] Moloney J.N., Cotter T.G. (2018). ROS signalling in the biology of cancer. Semin. Cell Dev. Biol..

[42] Das A. (2023). The emerging role of microplastics in systemic toxicity: Involvement of reactive oxygen species (ROS). Sci. Total Environ..

[43] Hajam Y.A., Rani R., Ganie S.Y., Sheikh T.A., Javaid D., Qadri S.S., Pramodh S., Alsulimani A., Alkhanani M.F., Harakeh S. (2022). Oxidative Stress in Human Pathology and Aging: Molecular Mechanisms and Perspectives. Cells.

[44] Camargo L.L., Wang Y., Rios F.J., McBride M., Montezano A.C., Touyz R.M. (2023). Oxidative Stress and Endoplasmic Reticular Stress Interplay in the Vasculopathy of Hypertension. Can. J. Cardiol..

[45] Hawkins C.L., Davies M.J. (2019). Detection, identification, and quantification of oxidative protein modifications. J. Biol. Chem..

[46] Valgimigli L. (2023). Lipid Peroxidation and Antioxidant Protection. Biomolecules.

[47] May-Zhang L.S., Kirabo A., Huang J., Linton M.F., Davies S.S., Murray K.T. (2021). Scavenging Reactive Lipids to Prevent Oxidative Injury. Annu. Rev. Pharmacol. Toxicol..

[48] Jelic M.D., Mandic A.D., Maricic S.M., Srdjenovic B.U. (2021). Oxidative stress and its role in cancer. J. Cancer Res. Ther..

[49] Saed G.M., Diamond M.P., Fletcher N.M. (2017). Updates of the role of oxidative stress in the pathogenesis of ovarian cancer. Gynecol. Oncol..

[50] Kang C., Ren X., Lee D., Ramesh R., Nimmo S., Yang-Hartwich Y., Kim D. (2024). Harnessing small extracellular vesicles for pro-oxidant delivery: Novel approach for drug-sensitive and resistant cancer therapy. J. Control. Release.

[51] Fletcher J.I., Williams R.T., Henderson M.J., Norris M.D., Haber M. (2016). ABC transporters as mediators of drug resistance and contributors to cancer cell biology. Drug Resist. Updat..

[52] Chen F.Q., Zhang J.M., Fang X.F., Yu H., Liu Y.L., Li H., Wang Y.T., Chen M.W. (2017). Reversal of paclitaxel resistance in human ovarian cancer cells with redox-responsive micelles consisting of α-tocopheryl succinate-based polyphosphoester copolymers. Acta Pharmacol. Sin..

[53] Gana C.C., Hanssen K.M., Yu D.M.T., Flemming C.L., Wheatley M.S., Conseil G., Cole S.P.C., Norris M.D., Haber M., Fletcher J.I. (2019). MRP1 modulators synergize with buthionine sulfoximine to exploit collateral sensitivity and selectively kill MRP1-expressing cancer cells. Biochem. Pharmacol..

[54] Mann E.K., Lee K.J., Chen D., da Silva L.M., Dal Zotto V.L., Scalici J., Gassman N.R. (2021). Associations between DNA Damage and PD-L1 Expression in Ovarian Cancer, a Potential Biomarker for Clinical Response. Biology.

[55] Xie F., Guo W., Wang X., Zhou K., Guo S., Liu Y., Sun T., Li S., Xu Z., Yuan Q. (2024). Mutational profiling of mitochondrial DNA reveals an epithelial ovarian cancer-specific evolutionary pattern contributing to high oxidative metabolism. Clin. Transl. Med..

[56] Mishra B., Lawson G.W., Ripperdan R., Ortiz L., Luderer U. (2018). Charged-Iron-Particles Found in Galactic Cosmic Rays are Potent Inducers of Epithelial Ovarian Tumors. Radiat. Res..

[57] Cuzziol C.I., Castanhole-Nunes M.M.U., Pavarino É.C., Goloni-Bertollo E.M. (2020). MicroRNAs as regulators of VEGFA and NFE2L2 in cancer. Gene.

[58] Lu T.X., Rothenberg M.E. (2018). MicroRNA. J. Allergy Clin. Immunol..

[59] Acuña S.M., Floeter-Winter L.M., Muxel S.M. (2020). MicroRNAs: Biological Regulators in Pathogen–Host Interactions. Cells.

[60] Marí-Alexandre J., Carcelén A.P., Agababyan C., Moreno-Manuel A., García-Oms J., Calabuig-Fariñas S., Gilabert-Estellés J. (2019). Interplay Between MicroRNAs and Oxidative Stress in Ovarian Conditions with a Focus on Ovarian Cancer and Endometriosis. Int. J. Mol. Sci..

[61] Zenkov N.K., Kozhin P.M., Chechushkov A.V., Martinovich G.G., Kandalintseva N.V., Menshchikova E.B. (2017). Mazes of Nrf2 Regulation. Biochemistry.

[62] Lu M.C., Ji J.A., Jiang Z.Y., You Q.D. (2016). The Keap1-Nrf2-ARE Pathway As a Potential Preventive and Therapeutic Target: An Update. Med. Res. Rev..

[63] Liu S., Pi J., Zhang Q. (2022). Signal amplification in the KEAP1-NRF2-ARE antioxidant response pathway. Redox Biol..

[64] Krajka-Kuźniak V., Paluszczak J., Baer-Dubowska W. (2017). The Nrf2-ARE signaling pathway: An update on its regulation and possible role in cancer prevention and treatment. Pharmacol. Rep..

[65] Chen N., Hu M., Jiang T., Xiao P., Duan J.A. (2024). Insights into the molecular mechanisms, structure-activity relationships and application prospects of polysaccharides by regulating Nrf2-mediated antioxidant response. Carbohydr. Polym..

[66] Li D., Hong X., Zhao F., Ci X., Zhang S. (2021). Targeting Nrf2 may reverse the drug resistance in ovarian cancer. Cancer Cell Int..

[67] Xia M., Yu H., Gu S., Xu Y., Su J., Li H., Kang J., Cui M. (2014). p62/SQSTM1 is involved in cisplatin resistance in human ovarian cancer cells via the Keap1-Nrf2-ARE system. Int. J. Oncol..

[68] Xia M., Yan X., Zhou L., Xu L., Zhang L., Yi H., Su J. (2020). p62 Suppressed VK3-induced Oxidative Damage Through Keap1/Nrf2 Pathway In Human Ovarian Cancer Cells. J. Cancer.

[69] Tossetta G., Fantone S., Montanari E., Marzioni D., Goteri G. (2022). Role of NRF2 in Ovarian Cancer. Antioxidants.

[70] Gasparri M.L., Bardhi E., Ruscito I., Papadia A., Farooqi A.A., Marchetti C., Bogani G., Ceccacci I., Mueller M.D., Benedetti Panici P. (2017). PI3K/AKT/mTOR Pathway in Ovarian Cancer Treatment: Are We on the Right Track?. Geburtshilfe Frauenheilkd.

[71] Tewari D. (2022). Natural products targeting the PI3K-Akt-mTOR signaling pathway in cancer: A novel therapeutic strategy. Semin. Cancer Biol..

[72] Mabuchi S., Kuroda H., Takahashi R., Sasano T. (2015). The PI3K/AKT/mTOR pathway as a therapeutic target in ovarian cancer. Gynecol. Oncol..

[73] Liu L.-Z., Hu X.-W., Xia C., He J., Zhou Q., Shi X., Fang J., Jiang B.-H. (2006). Reactive oxygen species regulate epidermal growth factor-induced vascular endothelial growth factor and hypoxia-inducible factor-1α expression through activation of AKT and P70S6K1 in human ovarian cancer cells. Free. Radic. Biol. Med..

[74] Ediriweera M.K., Tennekoon K.H., Samarakoon S.R. (2019). Role of the PI3K/AKT/mTOR signaling pathway in ovarian cancer: Biological and therapeutic significance. Semin. Cancer Biol..

[75] Ghoneum A., Said N. (2019). PI3K-AKT-mTOR and NFκB Pathways in Ovarian Cancer: Implications for Targeted Therapeutics. Cancers.

[76] Xu L., Wang L., Gan Y., Lin J., Ning S., Deng J., Ning Y., Feng W. (2024). Interference with ANXA8 inhibits the malignant progression of ovarian cancer by suppressing the activation of the Wnt/β-catenin signaling pathway via UCHL5. Aging.

[77] Koni M., Pinnarò V., Brizzi M.F. (2020). The Wnt Signalling Pathway: A Tailored Target in Cancer. Int. J. Mol. Sci..

[78] Teeuwssen M., Fodde R. (2019). Wnt Signaling in Ovarian Cancer Stemness, EMT, and Therapy Resistance. J. Clin. Med..

[79] Ji J., Qian Q., Cheng W., Ye X., Jing A., Ma S., Ding Y., Ma X., Wang Y., Sun Q. (2024). FOXP4-mediated induction of PTK7 activates the Wnt/β-catenin pathway and promotes ovarian cancer development. Cell Death Dis..

[80] Pei L., Zhao F., Zhang Y. (2024). USP43 impairs cisplatin sensitivity in epithelial ovarian cancer through HDAC2-dependent regulation of Wnt/β-catenin signaling pathway. Apoptosis.

[81] Karimaian A., Majidinia M., Bannazadeh Baghi H., Yousefi B. (2017). The crosstalk between Wnt/β-catenin signaling pathway with DNA damage response and oxidative stress: Implications in cancer therapy. DNA Repair.

[82] Akbarzadeh M., Akbarzadeh S., Majidinia M. (2020). Targeting Notch signaling pathway as an effective strategy in overcoming drug resistance in ovarian cancer. Pathol. Res. Pract..

[83] Diao B., Sun C., Yu P., Zhao Z., Yang P. (2023). LAMA5 promotes cell proliferation and migration in ovarian cancer by activating Notch signaling pathway. FASEB J..

[84] Bocchicchio S., Tesone M., Irusta G. (2019). Convergence of Wnt and Notch signaling controls ovarian cancer cell survival. J. Cell. Physiol..

[85] Cai Q., Yang J., Shen H., Xu W. (2024). Cancer-associated adipocytes in the ovarian cancer microenvironment. Am. J. Cancer Res..

[86] Maj T., Wang W., Crespo J., Zhang H., Wang W., Wei S., Zhao L., Vatan L., Shao I., Szeliga W. (2017). Oxidative stress controls regulatory T cell apoptosis and suppressor activity and PD-L1-blockade resistance in tumor. Nat. Immunol..

[87] An Y., Yang Q. (2021). Tumor-associated macrophage-targeted therapeutics in ovarian cancer. Int. J. Cancer.

[88] Rasool M., Malik A., Basit Ashraf M.A., Parveen G., Iqbal S., Ali I., Qazi M.H., Asif M., Kamran K., Iqbal A. (2016). Evaluation of Matrix Metalloproteinases, Cytokines and Their Potential Role in the Development of Ovarian Cancer. PLoS ONE.

[89] El-Arabey A.A., Alkhalil S.S., Al-Shouli S.T., Awadalla M.E., Alhamdi H.W., Almanaa T.N., Mohamed S., Abdalla M. (2023). Revisiting macrophages in ovarian cancer microenvironment: Development, function and interaction. Med. Oncol..

[90] Oršolić N., Kunštić M., Kukolj M., Gračan R., Nemrava J. (2016). Oxidative stress, polarization of macrophages and tumour angiogenesis: Efficacy of caffeic acid. Chem. Biol. Interact..

[91] Kumar S., Mittal S., Gupta P., Singh M., Chaluvally-Raghavan P., Pradeep S. (2022). Metabolic Reprogramming in Tumor-Associated Macrophages in the Ovarian Tumor Microenvironment. Cancers.

[92] Pankowska K.A., Będkowska G.E., Chociej-Stypułkowska J., Rusak M., Dąbrowska M., Osada J. (2023). Crosstalk of Immune Cells and Platelets in an Ovarian Cancer Microenvironment and Their Prognostic Significance. Int. J. Mol. Sci..

[93] Worzfeld T., Pogge Von Strandmann E., Huber M., Adhikary T., Wagner U., Reinartz S., Müller R. (2017). The Unique Molecular and Cellular Microenvironment of Ovarian Cancer. Front. Oncol..

[94] Mikuła-Pietrasik J., Uruski P., Szubert S., Szpurek D., Sajdak S., Tykarski A., Książek K. (2017). Malignant ascites determine the transmesothelial invasion of ovarian cancer cells. Int. J. Biochem. Cell Biol..

[95] McQuade R.M., Stojanovska V., Bornstein J.C. (2018). PARP inhibition in platinum-based chemotherapy: Chemopotentiation and neuroprotection. Pharmacol. Res..

[96] Kanakkanthara A., Hou X., Ekstrom T.L., Zanfagnin V., Huehls A.M., Kelly R.L., Ding H., Larson M.C., Vasmatzis G., Oberg A.L. (2022). Repurposing Ceritinib Induces DNA Damage and Enhances PARP Inhibitor Responses in High-Grade Serous Ovarian Carcinoma. Cancer Res..

[97] Wang Y.Y., Chen Y.K., Hu S.C., Hsu Y.L., Tsai C.H., Chi T.C., Huang W.L., Hsieh P.W., Yuan S.F. (2017). CYT-Rx20 inhibits ovarian cancer cells in vitro and in vivo through oxidative stress-induced DNA damage and cell apoptosis. Cancer Chemother. Pharmacol..

[98] Hou D., Xu G., Zhang C., Li B., Qin J., Hao X., Liu Q., Zhang X., Liu J., Wei J. (2017). Berberine induces oxidative DNA damage and impairs homologous recombination repair in ovarian cancer cells to confer increased sensitivity to PARP inhibition. Cell Death Dis..

[99] Aleissa M.S., Al-Zharani M., Alneghery L.M., Aleissa A.M. (2023). Berberine enhances the sensitivity of radiotherapy in ovarian cancer cell line (SKOV-3). Saudi Pharm. J..

[100] Sahai R., Bhattacharjee A., Shukla V.N., Yadav P., Hasanain M., Sarkar J., Narender T., Mitra K. (2020). Gedunin isolated from the mangrove plant Xylocarpus granatum exerts its anti-proliferative activity in ovarian cancer cells through G2/M-phase arrest and oxidative stress-mediated intrinsic apoptosis. Apoptosis.

[101] Mbemi A.T., Sims J.N., Yedjou C.G., Noubissi F.K., Gomez C.R., Tchounwou P.B. (2020). Vernonia calvoana Shows Promise towards the Treatment of Ovarian Cancer. Int. J. Mol. Sci..

[102] Wu Q., Wei M., Yao L., Cheng X., Lu W., Xie X., Li X. (2022). Hyperthermia synergistically enhances antitumor efficacy of PARP inhibitor through impacting homologous recombination repair and oxidative stress in vitro. Biochem. Biophys. Res. Commun..

[103] Liu J., Han X., Zhang T., Tian K., Li Z., Luo F. (2023). Reactive oxygen species (ROS) scavenging biomaterials for anti-inflammatory diseases: From mechanism to therapy. J. Hematol. Oncol..

[104] Saha S., Saso L., Armagan G. (2023). Cancer Prevention and Therapy by Targeting Oxidative Stress Pathways. Molecules.

[105] Ali S.S., Ahsan H., Zia M.K., Siddiqui T., Khan F.H. (2020). Understanding oxidants and antioxidants: Classical team with new players. J. Food Biochem..

[106] Halliwell B. (2024). Understanding mechanisms of antioxidant action in health and disease. Nat. Rev. Mol. Cell Biol..

[107] Demirci-Çekiç S., Özkan G., Avan A.N., Uzunboy S., Çapanoğlu E., Apak R. (2022). Biomarkers of Oxidative Stress and Antioxidant Defense. J. Pharm. Biomed. Anal..

[108] Milani A., Basirnejad M., Shahbazi S., Bolhassani A. (2017). Carotenoids: Biochemistry, pharmacology and treatment. Br. J. Pharmacol..

[109] Gulcin İ. (2020). Antioxidants and antioxidant methods: An updated overview. Arch. Toxicol..

[110] Kaźmierczak-Barańska J., Boguszewska K., Adamus-Grabicka A., Karwowski B.T. (2020). Two Faces of Vitamin C-Antioxidative and Pro-Oxidative Agent. Nutrients.

[111] Harris I.S., DeNicola G.M. (2020). The Complex Interplay between Antioxidants and ROS in Cancer. Trends Cell Biol..

[112] Bansal A., Simon M.C. (2018). Glutathione metabolism in cancer progression and treatment resistance. J. Cell Biol..

[113] Asantewaa G., Harris I.S. (2021). Glutathione and its precursors in cancer. Curr. Opin. Biotechnol..

[114] Poljsak B., Milisav I. (2018). The Role of Antioxidants in Cancer, Friends or Foes?. Curr. Pharm. Des..

[115] Xu J., Li Y., Hu H. (2019). Effects of lycopene on ovarian cancer cell line SKOV3 in vitro: Suppressed proliferation and enhanced apoptosis. Mol. Cell. Probes.

[116] Holzapfel N.P., Shokoohmand A., Wagner F., Landgraf M., Champ S., Holzapfel B.M., Clements J.A., Hutmacher D.W., Loessner D. (2017). Lycopene reduces ovarian tumor growth and intraperitoneal metastatic load. Am. J. Cancer Res..

[117] Xu Y., Guo X., Wang G., Zhou C. (2020). Vitamin C Inhibits Metastasis of Peritoneal Tumors By Preventing Spheroid Formation in ID8 Murine Epithelial Peritoneal Cancer Model. Front. Pharmacol..

[118] Markowska A., Antoszczak M., Markowska J., Huczyński A. (2022). Role of Vitamin C in Selected Malignant Neoplasms in Women. Nutrients.

[119] Gregoraszczuk E.L., Zajda K., Tekla J., Respekta N., Zdybał P., Such A. (2021). Vitamin C supplementation had no side effect in non-cancer, but had anticancer properties in ovarian cancer cells. Int. J. Vitam. Nutr. Res..

[120] Sun M.H., Gong T.T., Xu H.L., Yin J.L., Yang H.J., Zou B.J., Chen H.Y., Du Z.D., Wang R., Yan S. (2023). Association between pre-diagnostic dietary antioxidant vitamin consumption and ovarian cancer survival: A prospective cohort study. Food Funct..

[121] Gui H., Jin Y., Lin A., Wang P., Wang Y., Zhu H. (2021). Rosmarinic acid relieves cisplatin-induced ovary toxicity in female mice via suppression of oxidative stress and inflammation. J. Biochem. Mol. Toxicol..

[122] Chen Y.P., Shih P.C., Feng C.W., Wu C.C., Tsui K.H., Lin Y.H., Kuo H.M., Wen Z.H. (2021). Pardaxin Activates Excessive Mitophagy and Mitochondria-Mediated Apoptosis in Human Ovarian Cancer by Inducing Reactive Oxygen Species. Antioxidants.

[123] Hou D., Liu Z., Xu X., Liu Q., Zhang X., Kong B., Wei J.J., Gong Y., Shao C. (2018). Increased oxidative stress mediates the antitumor effect of PARP inhibition in ovarian cancer. Redox Biol..

[124] Kim T.H., Park J.H., Woo J.S. (2019). Resveratrol induces cell death through ROS-dependent downregulation of Notch1/PTEN/Akt signaling in ovarian cancer cells. Mol. Med. Rep..

[125] Nirgude S., Choudhary B. (2021). Insights into the role of GPX3, a highly efficient plasma antioxidant, in cancer. Biochem. Pharmacol..

[126] Worley B.L., Kim Y.S., Mardini J., Zaman R., Leon K.E., Vallur P.G., Nduwumwami A., Warrick J.I., Timmins P.F., Kesterson J.P. (2019). GPx3 supports ovarian cancer progression by manipulating the extracellular redox environment. Redox Biol..

[127] Geng D., Zhou Y., Wang M. (2024). Advances in the role of GPX3 in ovarian cancer (Review). Int. J. Oncol..

[128] Hemachandra L.P.M.P., Shin D.-H., Dier U., Iuliano J.N., Engelberth S.A., Uusitalo L.M., Murphy S.K., Hempel N. (2015). Mitochondrial Superoxide Dismutase Has a Protumorigenic Role in Ovarian Clear Cell Carcinoma. Cancer Res..

[129] Chiappetta G., Gamberi T., Faienza F., Limaj X., Rizza S., Messori L., Filomeni G., Modesti A., Vinh J. (2022). Redox proteome analysis of auranofin exposed ovarian cancer cells (A2780). Redox Biol..

[130] Oommen D., Yiannakis D., Jha A.N. (2016). BRCA1 deficiency increases the sensitivity of ovarian cancer cells to auranofin. Mutat. Res. Mol. Mech. Mutagen..

[131] Bae I., Fan S., Meng Q., Rih J.K., Kim H.J., Kang H.J., Xu J., Goldberg I.D., Jaiswal A.K., Rosen E.M. (2004). BRCA1 Induces Antioxidant Gene Expression and Resistance to Oxidative Stress. Cancer Res..

[132] Kobayashi H., Imanaka S., Shigetomi H. (2022). Revisiting therapeutic strategies for ovarian cancer by focusing on redox homeostasis (Review). Oncol. Lett..

[133] Zuo C., Cao H., Song Y., Gu Z., Huang Y., Yang Y., Miao J., Zhu L., Chen J., Jiang Y. (2022). Nrf2: An all-rounder in depression. Redox Biol..

[134] Yan R., Lin B., Jin W., Tang L., Hu S., Cai R. (2023). NRF2, a Superstar of Ferroptosis. Antioxidants.

[135] Tossetta G., Marzioni D. (2022). Natural and synthetic compounds in Ovarian Cancer: A focus on NRF2/KEAP1 pathway. Pharmacol. Res..

[136] Kitamura H., Motohashi H. (2018). NRF2 addiction in cancer cells. Cancer Sci..

[137] Ishimoto T., Nagano O., Yae T., Tamada M., Motohara T., Oshima H., Oshima M., Ikeda T., Asaba R., Yagi H. (2011). CD44 Variant Regulates Redox Status in Cancer Cells by Stabilizing the xCT Subunit of System xc− and Thereby Promotes Tumor Growth. Cancer Cell.

[138] Mvunta D.H., Miyamoto T., Asaka R., Yamada Y., Ando H., Higuchi S., Ida K., Kashima H., Shiozawa T. (2017). SIRT1 Regulates the Chemoresistance and Invasiveness of Ovarian Carcinoma Cells. Transl. Oncol..

[139] Chen Y.J., Yuan C.C., Chow K.C., Wang P.H., Lai C.R., Yen M.S., Wang L.S. (2005). Overexpression of dihydrodiol dehydrogenase is associated with cisplatin-based chemotherapy resistance in ovarian cancer patients. Gynecol. Oncol..

[140] Deng H.B., Parekh H.K., Chow K.C., Simpkins H. (2002). Increased expression of dihydrodiol dehydrogenase induces resistance to cisplatin in human ovarian carcinoma cells. J. Biol. Chem..

[141] Deng H.B., Adikari M., Parekh H.K., Simpkins H. (2004). Ubiquitous induction of resistance to platinum drugs in human ovarian, cervical, germ-cell and lung carcinoma tumor cells overexpressing isoforms 1 and 2 of dihydrodiol dehydrogenase. Cancer Chemother. Pharmacol..

[142] Chen J., Adikari M., Pallai R., Parekh H.K., Simpkins H. (2008). Dihydrodiol dehydrogenases regulate the generation of reactive oxygen species and the development of cisplatin resistance in human ovarian carcinoma cells. Cancer Chemother. Pharmacol..

[143] Choi J.-A., Lee E.H., Cho H., Kim J.H. (2023). High-Dose Selenium Induces Ferroptotic Cell Death in Ovarian Cancer. Int. J. Mol. Sci..

[144] Parish M., Massoud G., Hazimeh D., Segars J., Islam M.S. (2023). Green Tea in Reproductive Cancers: Could Treatment Be as Simple?. Cancers.

[145] Xu Y.Q., Gao Y., Granato D. (2021). Effects of epigallocatechin gallate, epigallocatechin and epicatechin gallate on the chemical and cell-based antioxidant activity, sensory properties, and cytotoxicity of a catechin-free model beverage. Food Chem..

[146] Włodarczyk M., Ciebiera M., Nowicka G., Łoziński T., Ali M., Al-Hendy A. (2024). Epigallocatechin Gallate for the Treatment of Benign and Malignant Gynecological Diseases-Focus on Epigenetic Mechanisms. Nutrients.

[147] Akbarzadeh M., Movassaghpour A.A., Ghanbari H., Kheirandish M., Fathi Maroufi N., Rahbarghazi R., Nouri M., Samadi N. (2017). The potential therapeutic effect of melatonin on human ovarian cancer by inhibition of invasion and migration of cancer stem cells. Sci. Rep..

[148] Targhazeh N., Reiter R.J., Rahimi M., Qujeq D., Yousefi T., Shahavi M.H., Mir S.M. (2022). Oncostatic activities of melatonin: Roles in cell cycle, apoptosis, and autophagy. Biochimie.

[149] Menéndez-Menéndez J., Martínez-Campa C. (2018). Melatonin: An Anti-Tumor Agent in Hormone-Dependent Cancers. Int. J. Endocrinol..

[150] Tan D.X., Manchester L.C., Esteban-Zubero E., Zhou Z., Reiter R.J. (2015). Melatonin as a Potent and Inducible Endogenous Antioxidant: Synthesis and Metabolism. Molecules.

[151] Chuffa L.G., Alves M.S., Martinez M., Camargo I.C., Pinheiro P.F., Domeniconi R.F., Júnior L.A., Martinez F.E. (2016). Apoptosis is triggered by melatonin in an in vivo model of ovarian carcinoma. Endocr. Relat. Cancer.

[152] Gurunathan S., Qasim M., Kang M.H., Kim J.H. (2021). Role and Therapeutic Potential of Melatonin in Various Type of Cancers. Onco Targets Ther..

[153] Terry P.D., Qin B., Camacho F., Moorman P.G., Alberg A.J., Barnholtz-Sloan J.S., Bondy M., Cote M.L., Funkhouser E., Guertin K.A. (2017). Supplemental Selenium May Decrease Ovarian Cancer Risk in African-American Women. J. Nutr..

[154] Wu S.X., Xiong R.G., Huang S.Y., Zhou D.D., Saimaiti A., Zhao C.N., Shang A., Zhang Y.J., Gan R.Y., Li H.B. (2023). Effects and mechanisms of resveratrol for prevention and management of cancers: An updated review. Crit. Rev. Food Sci. Nutr..

[155] Mozafaryan M.J., Rezaei P., Masoudpoor F., Bahri A., Samini M., Farkhondeh T., Pourhanifeh M.H., Samarghandian S. (2024). Resveratrol in the Treatment of Gynecological Cancer: Mechanisms and Therapeutic Potential. Curr. Med. Chem..

[156] Xu X.L., Deng S.L., Lian Z.X., Yu K. (2021). Resveratrol Targets a Variety of Oncogenic and Oncosuppressive Signaling for Ovarian Cancer Prevention and Treatment. Antioxidants.

[157] Liu X., Qi M., Li X., Wang J., Wang M. (2023). Curcumin: A natural organic component that plays a multi-faceted role in ovarian cancer. J. Ovarian Res..

[158] Kalinina E.V., Hasan A.A.S., Tatarskiy V.V., Volodina Y.L., Petrova A.S., Novichkova M.D., Zhdanov D.D., Nurmuradov N.K., Chernov N.N., Shtil A.A. (2022). Suppression of PI3K/Akt/mTOR Signaling Pathway and Antioxidant System and Reversal of Cancer Cells Resistance to Cisplatin under the Effect of Curcumin. Bull. Exp. Biol. Med..

[159] Prasad S., Gupta S.C., Tyagi A.K. (2017). Reactive oxygen species (ROS) and cancer: Role of antioxidative nutraceuticals. Cancer Lett..

[160] Shen X., Wang J., Deng B., Zhao Z., Chen S., Kong W., Zhou C., Bae-Jump V. (2024). Review of the Potential Role of Ascorbate in the Prevention and Treatment of Gynecological Cancers. Antioxidants.

[161] Koushik A., Wang M., Anderson K.E., van den Brandt P., Clendenen T.V., Eliassen A.H., Freudenheim J.L., Genkinger J.M., Håkansson N., Marshall J.R. (2015). Intake of vitamins A, C, and E and folate and the risk of ovarian cancer in a pooled analysis of 10 cohort studies. Cancer Causes Control..

[162] Long Y., Fei H., Xu S., Wen J., Ye L., Su Z. (2020). Association about dietary vitamin C intake on the risk of ovarian cancer: A meta-analysis. Biosci. Rep..

[163] Fairfield K.M., Hankinson S.E., Rosner B.A., Hunter D.J., Colditz G.A., Willett W.C. (2001). Risk of ovarian carcinoma and consumption of vitamins A, C, and E and specific carotenoids: A prospective analysis. Cancer.

